# Stress testing journals: a quasi-experimental study of rejection rates of a previously published paper

**DOI:** 10.1186/s12916-020-01550-9

**Published:** 2020-04-21

**Authors:** Kelly D. Cobey, Danielle B. Rice, Manoj M. Lalu, Daniel Abramowitz, Nadera Ahmadzai, Heather Cunningham, Ana Patricia Ayala, Hana Raffoul, Faizan Khan, Larissa Shamseer, David Moher

**Affiliations:** 1 Centre for Journalology, Clinical Epidemiology Program, The Ottawa Hospital Research Institute, Ottawa, Canada; 2 School of Epidemiology and Public Health, Faculty of Medicine, University of Ottawa, Ottawa, Canada; 3 Department of Psychology, McGill University, Montreal, Quebec Canada; 4 Department of Anesthesiology and Pain Medicine, The Ottawa Hospital, University of Ottawa, Ottawa, Canada; 5 Regenerative Medicine Program, The Ottawa Hospital, Ottawa, Canada; 6 Division of General Surgery, University of Toronto, Toronto, Canada; 7 Gerstein Science Information Centre, University of Toronto, Toronto, Canada; 8 Faculty of Engineering, University of Waterloo, Waterloo, Canada

**Keywords:** Scholarly publishing models, Open access, Predatory journals, Plagiarism

## Abstract

**Background:**

When a journal receives a duplicate publication, the ability to identify the submitted work as previously published, and reject it, is an assay to publication ethics best practices. The aim of this study was to evaluate how three different types of journals, namely open access (OA) journals, subscription-based journals, and presumed predatory journals, responded to receiving a previously published manuscript for review.

**Methods:**

We performed a quasi-experimental study in which we submitted a previously published article to a random sample of 602 biomedical journals, roughly 200 journals from each journal type sampled: OA journals, subscription-based journals, and presumed predatory journals. Three hundred and three journals received a Word version in manuscript format, while 299 journals received the formatted publisher’s PDF version of the published article. We then recorded responses to the submission received after approximately 1 month. Responses were reviewed, extracted, and coded in duplicate. Our primary outcome was the rate of rejection of the two types of submitted articles (PDF vs Word) within our three journal types.

**Results:**

We received correspondence back from 308 (51.1%) journals within our study timeline (32 days); (*N* = 46 predatory journals, *N* = 127 OA journals, *N* = 135 subscription-based journals). Of the journals that responded, 153 received the Word version of the paper, while 155 received the PDF version. Four journals (1.3%) accepted our paper, 291 (94.5%) journals rejected the paper, and 13 (4.2%) requested a revision. A chi-square test looking at journal type, and submission type, was significant (*χ*^2^ (4) = 23.50, *p* < 0.001). All four responses to accept our article came from presumed predatory journals, 3 of which received the Word format and 1 that received the PDF format. Less than half of journals that rejected our submissions did so because they identified ethical issues such as plagiarism with the manuscript (133 (45.7%)).

**Conclusion:**

Few journals accepted our submitted paper. However, our findings suggest that all three types of journals may not have adequate safeguards in place to recognize and act on plagiarism or duplicate submissions.

**Electronic supplementary material:**

**Supplementary information** accompanies this paper at 10.1186/s12916-020-01550-9.

## Background

Several spoof papers submitted to “predatory” journals have highlighted the self-interest these journals have: they seem to publish anything if authors are willing to pay an article processing fee. When spoof papers get accepted at predatory journals, they tend to attract a great deal of attention. Whether it is a Seinfeld-themed “case report” [1], or a bogus Star Wars themed paper with movie quotes [2], these papers make us laugh, but then encourage reflection on this darker side of publishing. They force us to acknowledge the potential threat predatory journals play in degrading the integrity of the open access publishing model and their impact on the scholarly literature more broadly.

Scientifically designed studies, which systematically evaluate predatory journals, are rare. Submitting a single low-quality paper to a journal, or a handful of journals, is relatively feasible to do. This however says little about the scale of the problem and does not allow us to track how predatory journals and legitimate journals have changed over time. The results of these spoof papers do inform us about the stability of particular journals to withstand worrisome publication practices. We can conceive of such studies as “stress tests” similar to what banks were required to withstand following the 2008 financial banking crisis. The first investigation to examine acceptance rates of spoof articles at a large group of journals was conducted in 2013, by journalist John Bohannon, and published by *Science Magazine* [3]. Bohannon submitted articles with obvious methodological and reporting issues, and made-up author and university names, to several presumed predatory journals and to several open access journals. In total he submitted versions of his flawed paper to 304 journals, 157 of which accepted his paper. Most journals that accepted his article were presumed to be predatory (69.42%; 84/121); however, many open access journals that were presumed not to be predatory also accepted the paper (38.32%; 64/167). Bohannon’s sting brought attention to the problem of predatory journals, but it also highlighted potential concerns with open access publishing and peer review more generally. The DOAJ (Directory of Open Access Journals) [4], which curates a list of legitimate open access journals, required all their journals to reapply for indexing as a consequence of this finding.

It has been more than 5 years since these data were collected. Bohannon, who is a journalist, did not design his study scientifically and therefore it had elements of bias in journal selection, in journal communication, and with lack of transparency in reporting.

No recent study auditing a broad range of journals with different publication models has been rigorously conducted. The number of predatory journals is growing [5]. Some federal agencies have created policies against predatory publishing [6, 7], and other stakeholder groups have launched awareness campaigns to educate researchers [8]. OMICS, a large and well-known predatory publisher and predatory conference organizer, was ordered by a federal judge in 2019 to pay a fine of more than 50 million dollars to resolve Federal Trade Commission charges in the USA, which found them guilty of making deceptive claims about their operations and fee structure [9]. It is unclear if these actions have influenced predatory journals, or if predatory journals have adapted during this timeframe.

Here, we describe the results of a quasi-experimental study in which we submitted a previously published article to more than 600 journals from three journal groups: presumed predatory journals, open access journals, and subscription-based journals. A previous study has found a substantial level of plagiarism within presumed predatory nursing journals [10]. We submitted a Microsoft Word version of the article to approximately half of these journals, and the publishers formatted PDF version to the other half. Our primary outcome was the rate of rejection of the previously published article at each of the journal types. We predicted that articles submitted to predatory journals would be rejected at a lower rate than articles submitted to open access or subscription-based journals, but that no journal type would be immune from accepting the article. As a secondary outcome, we looked at the type of editorial and peer review within the three journal types. We predicted that more of the subscription-based and open access journals, as compared to predatory journals, would immediately detect the paper as being plagiarized and reject it without sending it for peer review. We predicted that differences in rejection rates would be greatest when considering the PDF article submission as compared to the Word article submission.

## Methods

### Ethics, consent, and permissions

This study, which included deliberate deception and subsequent debriefing, received ethical approval from the Ottawa Health Science Network Research Ethics Board (REB #: 20180266-01H). We developed a study protocol and uploaded it to the Open Science Framework [11] prior to initiating data collection (See: https://osf.io/4ngk3/). Given the use of deception, participants (journal editorial team members) did not provide consent.

### Journal sampling

We sought to sample 200 journals from each of the three journal types, as described below. All journal titles selected for the sample were collated into an Excel file along with their respective URL. We checked for duplication of journals across our three randomly selected samples. We excluded all journals that indicated they only publish specific research designs, i.e., case studies, reviews, journals that specified submissions were by invite only, and journals that required a submission fee. We also excluded journals that did not have functioning websites (e.g., dead links, journal submission platforms not functioning), or journals that indicated they were no longer accepting manuscripts. Excluded journals were replaced with another randomly selected journal from the same group. Using a random number table generated in Excel, we randomly assigned half of the journals to be sent the Word version of the previously published article, and the other half the PDF version.

#### Potential “predatory” journals

We used an archived version (obtained from: https://beallslist.weebly.com/ on March 5, 2018) of Beall’s list of single journal publishers to identify predatory journals. We screened all journals on Beall’s List of single journal publishers independently in duplicate to identify biomedical journals that are currently active. We used the MEDLINE Journal Selection Criteria to define biomedical journals, namely journals that are “predominantly devoted to reporting original investigations in the biomedical and health sciences, including research in the basic sciences; clinical trials of therapeutic agents; effectiveness of diagnostic or therapeutic techniques; or studies relating to the behavioural, epidemiological, or educational aspects of medicine” [12]. We intended to select a random sample of 200 journals from those identified as biomedical; however, since there were only 158 biomedical journals on the archived version of Beall’s single journal publisher, as per our protocol, we randomly selected the remaining journals from the predatory publisher OMICS fleet of journals (*https://www.omicsonline.org/*). OMICS lists its journals by topic area; we selected all journals listed in their “Clinical & Medical Journal” section. Duplicate journals were removed. At the time of sampling, OMICS contained 500 “Clinical and Medical” journals. Using a random number table in Excel, we then randomly selected the number of journals remaining from the sample from these OMICS journals.

#### Presumed legitimate open access journals

We used PubMed Central [13], which is a free full-text archive of biomedical and life science journals managed by The National Library of Medicine’s National Center for Biotechnology Information, as the data source from which to extract biomedical open access journals. A CSV file of the list of journals was downloaded on April 5, 2018, which contained 3046 titles. The CSV file was opened in Excel and then sorted according to limit to full and immediate open access in order to eliminate hybrid subscription-open access journals. The list was further limited to filter out former journal titles based upon deposit status. This resulted in 1451 journals which combined both biomedical and life science journals. Subsequently, using a random numbers table in Excel, a sample of 200 journals was selected. The initial sample of OA journals included 82 unique publishers.

#### Presumed legitimate (primarily) subscription-based journals

Scopus is a multidisciplinary abstract and index database which indexes approximately 36,000 journal titles, both open access as well as subscription-based, from the life science, social sciences, physical sciences, and health sciences. The Scopus source title list, accurate as of October 2017, was downloaded on April 5, 2018, and sorted according to the following criteria: active (to removed ceased titles); source type (to remove book series and trade journals); open access (to remove open access journals); and filtered to the Health Sciences subheading which includes journals categorized by Scopus in the Medicine, Nursing, Dentistry, and Health Professions (to limit to biomedical journals).

The original Scopus title list contained 36,832 titles, and after application of filters to narrow to a subscription-based set of biomedical journals, the list contained 5716 titles. From this subset, 200 were selected by using Excel to generate a random number table. The initial sample of subscription-based journals included 71 unique publishers.

### Preparation of manuscript

The article we submitted was authored by members of this team. It was published in *Nature* in 2017 and it described epidemiological characteristics of predatory journals and their reporting quality [14]. Prior to conducting this study, we sought and obtained permission from *Nature* to use this publication for this purpose. Although there were several authors on the published article we submitted, to avoid any potential reputational damage to the broader team, only the primary investigator’s name (DM) was left on the submitted article. We digitally manipulated the PDF to remove all other author names. We prepared three versions of our article: (1) the accepted manuscript in Microsoft Word format; (2) the published PDF version of the article, including *Nature’s* formatting; (3) a Microsoft Word version that contained full page images of the published PDF version of the article, including *Nature’s* formatting. The latter of these versions was created to accommodate that many of the journal submission platforms would not accept PDF documents, both (2) and (3) were considered equivalently.

### Submission of article to journals

Members of the research team submitted articles (either PDF or Word) to all journals between May 7 and 22, 2018. Team members visited each of the selected journal websites to determine the appropriate method for submitting the work. If e-mail submissions were possible, the article was submitted via e-mail. E-mails were sent using MailMerge from the primary investigator (DM) using a standard submission e-mail template letter (see Additional file 1) and providing his genuine details for correspondence. If e-mail submissions were not standard protocol at the journal, we submitted via the journal’s online submission platform. Again, all submissions were ostensibly submitted from the corresponding author (DM) and provided his genuine details for correspondence.

As part of the online submissions, researchers often need to indicate information such as key words, the manuscript research area, and suggested reviewers. When such information was required to submit an article, the research team populated these fields using the most relevant content. Deception was used to indicate the paper was not previously published and to respond to all other online submission questions required to move forward with submission. Where possible, we used standard responses that we developed (see examples in Additional file 2).

### Submission outcomes

We recorded the number of articles successfully submitted, and the date and format in which they were submitted (e-mail or via a submission platform). We then tracked all e-mail correspondence from each journal. When journals responded to the correspondence, we saved their e-mail response and uploaded it to DistillerSR (Evidence Partners, Ottawa, Canada) [15]. This is an auditable, cloud-based software used to assist with data extraction. Two members of the research team independently extracted information from the e-mails received, using a piloted extraction form. Discrepancies were resolved through a third independent reviewer. We extracted the e-mail address from which correspondence was received, the date of correspondence and whether this was within our study timeline, whether an editorial decision was made on the submitted paper, and if so, what the decision was.

The frequency and types of initial decisions with respect to our submitted paper at each journal were coded according to the categories in Table 1, with the option to select more than one response.

**Table 1:** Categories used to code e-mail responses from journals

1. Rejected without external peer review by editorial staff due to ethical concerns (e.g., editor note they detected the duplicate publication or plagiarism)	
2. Rejected without external peer review by editorial staff due to topic/scope of article being outside journal scope	
3. Rejected without peer review by the editorial staff for any other reason (reasons to be specified)	
4. Sent out for external peer review, rejected due to ethical concerns (e.g., duplicate publication, plagiarism)	
5. Sent out for external peer review, rejected due to methodological concerns	
6. Sent out for external peer review, revision invited but ethical concerns noted	
7. Sent out for external peer review, revision invited without ethics concerns noted	
8. Sent out for external peer review, accepted with extremely minor or no revisions	
9. Accepted immediately without having undergone external peer review (i.e., no external peer review comments appended to the editors decision)	
10. Provisional acceptance pending minor changes based on editorial and/or peer review comments.	
11. Other (to be specified)	
12. Sent out for external peer review, then rejected but no reason given	
13. Rejected, no reason given	

For the purpose of coding these e-mails, we defined peer review as any form of feedback or suggestions for changes to the manuscript. As noted above, we distinguished between review from the journal (i.e., editorial review) and review external to the journal (i.e., by research peers). Thirty-two days after the last article was submitted, all journals were sent a debrief form explaining the study aims and linking to our protocol and our ethics approval documentation. The letter specified the withdrawal of our submission and that we would neither complete any requested revisions, nor agree to publication of the work, nor proceed with payment of any requests for article processing charges (Additional file 3). A timeframe of approximately 1 month was selected for feasibility purposes.

### Analysis

Data analyses were carried out using IBM SPSS v 24 (NY, IBM). Our primary outcome was the rate of rejection of the two types of submitted articles (PDF vs Word) within each of the three journal types. The rate of rejection for the submitted articles is summarized below using frequencies and percentages. We tested for differences using a 3 × 2 chi-square test.

### Protocol amendments

There were a few instances in which we diverged from our planned protocol. Of the journals that were initially randomly sampled, we added the exclusion criteria that we would not submit to journals that indicate they only publish specific research (i.e., case studies, reviews; journals that specify submissions are by invite only) or to journals that required a submission fee. Any journals excluded for these reasons were replaced in the sample. Due to challenges experienced with required journal exclusions, and the dynamic nature of the study with multiple researchers submitting to our sampled journals simultaneously, these numbers varied slightly from the 200 per group we planned. The window of time we took to submit the research articles was slightly longer than the 1 week anticipated. We had planned to send the debrief form to journals in our sample 30 days after the last article was submitted but sent this 32 days later instead due to some challenges encountered with the MailMerge tool. We had created a series of categories to code responses from journals, numbered 1–11 above, but added additional categories (12–13 above) based on the correspondence we received. We did not repeat the analysis considering the type of article submitted (Word vs PDF) as planned, again due to low N. Further, we had not anticipated that some journals would correspond via E-mails more than once. In instances where this occurred, we coded the last E-mail sent, which was received prior to sending our debrief form, and that provided an editorial decision. Finally, based on correspondence during the study period, we modified our study debrief form (Additional file 3) slightly after ethical approval. This debrief was sent to all journals.

## Results

A preprint describing the results of this study was posted ahead of journal submission on the Open Science Framework (https://osf.io/kre6j/).

### Article submissions

From the group of 600 journals identified to sample, many journals had to be excluded and replaced. Some of the randomly selected replacement journals also required exclusion. In total, we attempted to submit to 706 journals, of which we excluded 104 journals. The most common reasons for exclusion were that the journal only accepted particular topics or article types (e.g., case reports), *N* = 35, 33.7%; the journal registration or submission platform did not work, *N* = 30, 28.8%; and the journal indicated it was no longer accepting submissions, *N* = 14, 13.5%. For full exclusions, see Table 2. We ultimately submitted the article to 602 journals (*N* = 201 predatory journals, *N* = 199 OA journals, *N* = 202 subscription-based journals).

**Table 2:** Frequencies of journal exclusions from sample

**Reason for exclusion**	***N***	**%**
Journal only accepts specific types or topics of articles (e.g., case reports, medical images, pilot studies)	35	33.7
Journal registration or submission portal do not work	30	28.8
Journal no longer accepting submissions	14	13.5
Journal website does not work	6	5.8
Journal requires a submission fee	5	4.8
Other	14	13.5

A total of 303 journals received the Word version of the paper, while the remaining 299 received the PDF version. We received correspondence back from 308 (51.2%) journals within our study timeline (*N* = 46 predatory journals, *N* = 127 OA journals, *N* = 135 subscription-based journals). Of the journals that responded, 153 received the Word version of the paper, while 155 received the PDF version (Please see Table 3).

**Table 3:** Frequencies of article submissions and responses by journal type and article condition

**Journal type**	**Number of submissions**			**Number of responses**		
	**Total**	**PDF**	**Word**	**Total (*N*(%))**	**PDF**	**Word**
Predatory	201	100	101	46	21	25
Open access (OA)	199	99	100	127	66	61
Subscription-based	202	100	102	135	68	67
**Total**	**602**	**299**	**303**	**308 (51.2)**	**155**	**153**

### Primary outcome

Four journals (1.3%) accepted our paper, 291 (94.5%) journals rejected the paper, and 13 (4.2%) requested a revision. The chi-square test was significant (*χ*^2^(4) = 23.50, *p* < 0.001), meaning the observed distribution of data did not match the expected distribution: all four responses to accept our article came from predatory journals. One predatory journal (0.3%), 6 (1.9%) open access journals, and 6 (1.9%) traditional subscription-based journals requested a revision to the paper. The remaining journals rejected the submission (*N* = 41 predatory journals, *N* = 121 OA journals, *N* = 129 subscription-based journals). We did not repeat the analysis considering the type of article submitted (Word vs PDF) given that the vast majority of articles were rejected.

### Secondary outcome

We examined the editorial decisions within each of the journal groups, and in consideration of the type of article (Word vs PDF) we sent to them. Full results are reported in Tables 3 and 4.

**Table 4:** Frequencies of editorial decisions by journal type and article condition

**Journal type**	**Accept**			**Revision**			**Reject**			**No response**		
	**Total**	**PDF**	**Word**	**Total**	**PDF**	**Word**	**Total**	**PDF**	**Word**	**Total**	**PDF**	**Word**
**Predatory**	4	1	3	1	0	1	41	20	21	155	79	76
**Open access (OA)**	0	0	0	6	3	3	121	63	58	72	33	39
**Subscription-based**	0	0	0	6	4	2	129	64	65	67	32	35

#### Acceptances

Four journals, all predatory, accepted the paper outright; 3 of these journals received the Word document while 1 received the PDF.

#### Rejections

Of the 291 (48.3%) journals that rejected the paper, just 133 (45.7%) rejected the paper without external review due to ethical concerns (e.g., duplicate publication or plagiarism). These 133 journals can be classed as passing our stress test, and effectively rejecting the paper for the appropriate reasons. From this group of 113 journals who rejected the paper, 49 received the Word version and 84 received the PDF. Slightly fewer journals (*N* = 118, 40.5%) indicated that the article was being rejected for being outside the scope of the journal it was submitted to, this number includes 47 journals who received the PDF of the article as compared to 71 who received the Word version. Full details of journal rejections by category are reported in Table 5.

**Table 5:** Frequencies of article decision by journal type and article submission type. Note that some decisions e-mails were coded as applying to more than one of the 13 decision category numbers

Decision	Decision details	Journal type								
		Predatory (***N***=46)			Open access (OA) (***N***=127)			Subscription-based (***N***=135)		
		Total	PDF ***N***=21	Word ***N***=25	Total	PDF ***N***=66	Word ***N***=61	Total	PDF ***N***=68	Word ***N***=67
**1.**	**Rejected without external peer review by editorial staff due to ethical concerns (e.g. editor note they detected the duplicate publication or plagiarism)**	21 (45.7)	13 (61.9)	8 (32.0)	51 (40.2)	31 (47.0)	20 (32.8)	61(45.2)	40 (58.8)	21(31.3)
**2.**	**Rejected without external peer review by editorial staff due to topic/scope of article being outside journal scope**	12 (26.1)	4(19.0)	8(32.0)	53 (41.7)	24 (36.4)	29 (47.5)	53 (39.3)	19 (27.9)	34(50.7)
**3.**	**Rejected without peer review by the editorial staff for any other reason**	7 (15.2)	5(23.8)	2(8.0)	17 (13.4)	7 (10.6)	10 (16.4)	20 (14.8)	10 (14.7)	10 (14.9)
**4.**	**Sent out for external peer review, rejected due to ethical concerns (e.g. duplicate publication, plagiarism)**	1 (2.2)	-	1(4.0)	1 (0.8)	1 (1.5)	-	-	-	-
**5.**	**Sent out for external peer review, rejected due to methodological concerns**	-	-	-	1 (0.8)	1 (1.5)	-	-	-	-
**6.**	**Sent out for external peer review, revision invited but ethical concerns noted**	-	-	-	2 (1.6)	1(1.5)	1(1.5)	2 (1.5)	2 (2.9)	-
**7.**	**Sent out for external peer review, revision invited without ethics concerns noted**	1 (2.2)	-	1(4.0)	4(3.1)	2(3.0)	2(3.3)	4(3.0)	2 (2.9)	2(3.0)
**8.**	**Sent out for external peer review, accepted with extremely minor or no revisions**	-	-	-	-	-	-	-	-	-
**9.**	**Accepted immediately without having undergone external peer review (i.e. no external peer review comments appended to the editors decision)**	4 (8.7)	1 (4.8)	3(12.0)	-	-	-	-	-	-
**10.**	**Provisional acceptance pending minor changes based on editorial and/or peer review comments.**	-	-	-	-	-	-	-	-	-
**11.**	**Other**	-	-	-	2 (1.6)	-	2 (3.3)	-	-	-
**12.**	**Sent out for external peer review, then rejected but no reason given**	-	-	-	1 (0.8)	1 (1.5)	-	-	-	-
**13.**	**Rejected, no reason given**	4 (8.7)	1 (4.8)	3(12.0)	19 (15.0)	9 (13.6)	10 (16.4)	15 (11.1)	8 (11.8)	7 (10.4)

#### Revisions

Thirteen (4.2%) of journals requested a revision of the article, 6 of these journals were sent the Word version of the article, and 7 the PDF. These revisions came from OA journals (*N* = 6), traditional journals (*N* = 6), and predatory journals (*N* = 1).

#### No response

Nearly half of the journals we submitted to did not respond within the 32-day study window (*N* = 294, 48.8%). From these non-responders, 144 received the PDF, while 150 received the Word version of our article. Predatory journals did not respond to 77.1% of submissions, open access journals did not respond to 36.2%, and subscription-based journals did not respond to 33.2%.

## Discussion

Our primary outcome in this quasi-experimental study was the rate of rejection of the previously published article at each of the journal types we submitted to. Just four journals accepted our article, all of which were presumed to be predatory, and only one of which was submitted in PDF format. The predatory journals in our sample accepted our problematic paper at much lower rates than predatory journals targeted by Bohannon’s sting in 2014 (69.4% VS < 9% of predatory journals who responded). It is possible that, due to potential legal concerns, predatory journals are less likely to re-publish text from a large, well-known publisher without permission, as compared to an otherwise problematic paper***.*** Contrary to our prediction, no open access journals or traditional subscription-based journals accepted our article. This is a positive sign; however, it must be interpreted cautiously given that many of the journals we submitted to did not respond at all, some invited revisions, while others rejected the paper without identifying ethical concerns with it. It is notable that predatory journals responded to our manuscript submissions at a much lower rate that either open access or subscription-based journals.

It is possible that some of the journals that rejected our article sent us a standard e-mail rejection, which did not note ethical concerns, even if the editor had expressed these. Other journals may have been suspicious and chose not to respond at all. Failing to act on a duplicate submission/plagiarism is a problematic practice. In our view, journals, especially those who are members of the Committee on Publication Ethics (COPE) [16], should consider acting explicitly when plagiarism or unethical conduct is identified. Moreover, as per COPE guidance, journal editors are encouraged to follow up with relevant stakeholders such as the research institution of the corresponding author [17]. During this study, only a very small number of editors contacted the institution of the corresponding author to express their concerns about the submission (e.g., writing to the Dean of Medicine of the corresponding author, or to the Research Institute’s Director, or research ethics board).

Another concerning outcome of this study is the rate of non-response to our submissions. All journals in our sample had several weeks to decide on our article, yet almost half of them failed to do so. It is possible that our article was out for review at some of these journals. We did not check submission platforms to see the status of our article. In our view, it is problematic that any legitimate journal would not be able to quickly discern issues with our article (e.g., out of journals scope, previously published), particularly when the Nature formatted PDF was submitted. Our study serves as a stress test for the three journal types. In our view, only those journals that received our article and promptly rejected it due to ethical concerns (e.g., duplicate publication, plagiarism) passed this test (133/602; meaning just 22.1% ‘passed’). The failure of many journals to respond highlights inefficiencies in the editorial system. The same concern exists for publishers. Some publishers received multiple simultaneous submissions of our article to different journals they operate. Despite this, few publishers identified the work had been submitted to more than one of their journals. One notable exception was a large open access publisher who reached out directly querying why they had received 17 submissions of the same article, which they recognized as having been previously published.

If all articles received were immediately screened using plagiarism text matching software, a tool many journals promote, they would be able to identify and reject papers like ours and reduce editorial staff time needed to read and review the article. We recognize use of plagiarism software can be costly and suspect journals vary in their access to it, and their timing of using it within the editorial processes. We received few requests to revise our article, and many of these were requests to revise and resubmit to other journals within the same publisher’s fleet. This too would presumably not have occurred if the article had been screened by plagiarism software.

Since the publication of Bohannon’s 2013 sting, the criteria and methods used to list journals in the DOAJ have changed [18]. The fact that none of the OA journals listed in DOAJ in our sample accepted the article suggests these changes may provide better safeguards. Our study differs from Bohannon’s study in two other key areas. Firstly, we have focused our research on biomedical journals only, so the conclusions of our work are most relevant within this discipline. Secondly, in our sting, we submitted a paper that has been published previously in a well-known journal, rather than submitting a fictitious paper. This allowed us to assay whether there are safeguards in place at each of the journals to detect duplicate publication and protect against copyright infringements. Arguably, our article, specifically the Word version, would be less outlandish than Bohannon’s article submissions.

One challenge of this work was to accurately and efficiently identify the lists of three different types of journals we sampled. To identify potential predatory journals, we used an archived version of Beall’s list. This methodology is sub-optimal for a number of reasons. Firstly, Beall has been criticized for his lack of transparency in curating his lists. It was not always clear what criteria he used to list journals, or how he identified journals to assess as potentially predatory in the first place. Secondly, given the fluid nature of predatory journals, lists of low-quality journals are unlikely to be current. This may also explain our low overall response rate from predatory journals, as some journals may no longer be active. Despite these concerns, we know of no obvious and pragmatic alternative method to identify large groups of potentially predatory journals. The only other widely known lists of presumed predatory journals, to our knowledge, are curated by the commercial company Cabells and are behind a paywall [19]. Relatedly, as articles published in predatory journals have appeared in PUBMED Central [20], it is possible that some of the journals captured in our sample of presumed legitimate open access journal were in fact listed on Beall’s list. Recent work has established a consensus definition of predatory journals [21]. This definition may be useful to validate samples of predatory journals in future research.

## Conclusion

The outcomes of this study are significant to track journal publishing practices over time. The findings may be relevant to a broad group of stakeholders including research authors, research institutions, research ethics boards, journals/publishers, funders, and patient/public groups [22]. Having a system of regular audit in place to track journal operations is critical to identifying shortcomings and driving improvements in the scholarly publishing landscape. Failure to ensure these safeguards increases the potential for low-quality work to be shared that pollutes the quality of the research literature. This situation may be particularly concerning within biomedical research fields, where patient care may be impacted when patients themselves, or their care providers, come across predatory research.

One potential implication from our findings is journals that have been labeled as predatory may have generally improved their practices: they accepted our PDF submission, which should be obviously problematic immediately to any viewer, at very low rates. Predatory journals may have evolved to measures taken by the scholarly community. Some journals may be situated in the gray area between legitimate journals and predatory journals. Ongoing audit is needed to track changes in predatory journal operations in order to understand how they operate. Audits of legitimate journals are also needed to ensure best practice standards are being met and to understand what supports journals need to ensure compliance to best practices.

## Supplementary information

Additional file 1 Cover letter provided during article submission.

Additional file 2 Standard responses given on online submission platform intake forms.

Additional file 3 Debrief from sent to journals.

## Data Availability

The dataset supporting the conclusions of this article is available in the Open Science Framework: https://osf.io/rmgcn/.
