# Supplementary material for: Stress testing journals: a quasi-experimental study of rejection rates of a previously published paper
Source: BMC Med. 2020 Apr 21;18:88. doi: 10.1186/s12916-020-01550-9 (PMC7171725; doi:10.1186/s12916-020-01550-9)
Supplement: Supplementary file 2 — Standard responses given on online submission platform intake forms. [file 12916_2020_1550_MOESM2_ESM.docx]

**Additional file 2.** Standard responses given on online submission platform intake forms (developed a priori for use when submitting our previously published paper)

*Note: If unanticipated fields are encountered during article submission the research team will correspond and agree to additional standard responses to be used consistently across submissions.*

**Current funding sources:**

Research Chair, University of Ottawa

**Keywords:**

Journalology, Publication Science, Peer Review, Scholarly Communications, Quality of Reporting, Publication Models, Medicine, Health.

**Manuscript Details:**

Number of tables: 0

Number of figures: 1

**Suggestions for editor:**

*Select first editor on journal list*

**Previous Journal Submission Information:**

This article has not been previously submitted to the journal.

**Policy Requirements:**

*We will affirm all publication policy statements we are presented with during the article submission process. Examples of such statements may include statements such as:*

I have read and understood the publication charge policy.

I confirm that the author of this submission has understood the journal’s licensing policy.

As corresponding author, I take responsibility for the affirmations regarding submission of this article (e.g., that it is not under consideration by another journal), the integrity of the data presented in the article, and the statements regarding compliance with institutional ethics.

**Ethical Requirement Statements:**

Ethics approval is not required for this manuscript as no human data is included.

**Trial Registration Information:**

Not applicable.

**Financial Disclosure of Authors:**

Dr. Moher reports having no conflicts of interest.

**Data Availability Statement:**

All data corresponding to the study will be made publicly available using the Open Science Framework.
