# Supplementary material for: Stress testing journals: a quasi-experimental study of rejection rates of a previously published paper
Source: BMC Med. 2020 Apr 21;18:88. doi: 10.1186/s12916-020-01550-9 (PMC7171725; doi:10.1186/s12916-020-01550-9)
Supplement: Supplementary file 3 — Debrief from sent to journals. [file 12916_2020_1550_MOESM3_ESM.docx]

**Additional file 3:** Debrief form sent to journals

Email Subject: Article withdrawal and study debrief form

Dear [Insert journal name or editor name here],

I recently submitted an article entitled “Stop this waste of people, animals and money” for publication consideration in your journal. The submission of this article was part of a research study being conducted at the Ottawa Hospital Research Institute. The aim of this study was to evaluate the rejection rates of a previously published paper in journals with different publication models (open access, subscription, and with questionable publication practices (i.e., potentially predatory journals) in order to evaluate editorial and peer review vetting processes.

Specifically, we submitted a previously published paper to a series of 600 randomly selected journals to determine the rate of rejection of this article at each of the journal types. A full protocol for this study, completed and locked prior to the conduct of the study, is available *[insert OSF link]* and information on ethics approval for the research, received prior to the conduct of the study, can be found *[here].*

If your journal has not already rejected this submission, I would like to withdraw my submitted article “Stop this waste of people, animals and money” from further consideration. Please confirm that the withdrawal process is complete. I will not complete any requested revisions, provide payment of any sort, or publish the article at your journal.

Sincerely,

David Moher
