# Supplementary material for: Stress testing journals: a quasi-experimental study of rejection rates of a previously published paper
Source: BMC Med. 2020 Apr 21;18:88. doi: 10.1186/s12916-020-01550-9 (PMC7171725; doi:10.1186/s12916-020-01550-9)
Supplement: Supplementary file 1 — Cover letter provided during article submission. [file 12916_2020_1550_MOESM1_ESM.docx]

**Additional file 1.** Cover letter provided during article submission

[Insert date here]

Dear [Insert journal name or editor name here],

I am writing to submit my manuscript entitled “Stop this waste of people, animals and money” for consideration to be published in your journal. Please see attached.

There have been a number of rapid changes within the biomedical scholarly publishing landscape recently. This paper reports on a cross-sectional study that explores some of these recent changes. Changes to how biomedical research is produced and disseminated have the ability to directly impact patient care.

I believe this work will be of particular relevance to the readers of this journal given that the study findings are discussed in terms of their impact for relevant stakeholders including journals, publishers, researchers, funders, and patients.

I am sole author of this work and it is not currently under consideration elsewhere. I look forward to hearing from you.

Sincerely,

David Moher
